# FOXO4-DRI alleviates age-related testosterone secretion insufficiency by targeting senescent Leydig cells in aged mice

**DOI:** 10.18632/aging.102682

**Published:** 2020-01-20

**Authors:** Chi Zhang, Yun Xie, Haicheng Chen, Linyan Lv, Jiahui Yao, Min Zhang, Kai Xia, Xin Feng, Yanqing Li, Xiaoyan Liang, Xiangzhou Sun, Chunhua Deng, Guihua Liu

**Affiliations:** 1 Department of Andrology, The First Affiliated Hospital of Sun Yat-sen University, Guangzhou 510000, China; 2 Reproductive Medicine Research Center, The Sixth Affiliated Hospital of Sun Yat-sen University, Guangzhou 510000, China; 3 Guangdong Provincial Key Laboratory of Orthopedics and Traumatology, Guangzhou 510000, China; 4 Guangdong Provincial Key Laboratory of Colorectal and Pelvic Floor Diseases, Guangzhou 510000, China

**Keywords:** FOXO4-DRI, Leydig cell, senescence, male late-onset hypogonadism, senolytics

## Abstract

Male late-onset hypogonadism is an age-related disease, the core mechanism of which is dysfunction of senescent Leydig cells. Recent studies have shown that elimination of senescent cells can restore proper homeostasis to aging tissue. In the present study, we found that the fork head box O (FOXO) transcription factor FOXO4 was specially expressed in human Leydig cells and that its translocation to the nucleus in the elderly was related to decreased testosterone synthesis. Using hydrogen peroxide-induced senescent TM3 Leydig cells as an *in vitro* model, we observed that FOXO4 maintains the viability of senescent Leydig cells and suppresses their apoptosis. By disrupting the FOXO4-p53 interaction, FOXO4-DRI, a specific FOXO4 blocker, selectively induced p53 nuclear exclusion and apoptosis in senescent Leydig cells. In naturally aged mice, FOXO4-DRI improved the testicular microenvironment and alleviated age-related testosterone secretion insufficiency. These findings reveal the therapeutic potential of FOXO4-DRI for the treatment of male late-onset hypogonadism.

## INTRODUCTION

Male late-onset hypogonadism (LOH) is a clinical and biochemical syndrome associated with advancing age. It is characterized by a deficiency in serum testosterone levels and symptoms such as low sexual desire, erectile dysfunction, muscle mass loss, obesity, osteoporosis, and depression [1–3]. LOH reportedly affects 7-30% of aged males [4] and may be a significant detriment to their quality of life, adversely affecting the function of multiple organ systems [5]. Indeed, LOH is thought to be related to substantially higher risks of all-cause and cardiovascular mortality in aged males [6]. Leydig cells are the primary source of testosterone [7]. With aging, the number of Leydig cells per testis remains unchanged, but these cells may exhibit a reduced capacity for testosterone synthesis, resulting in an age-related decline of serum testosterone levels [8, 9]. These dysfunctional senescent Leydig cells play a core role in the pathogenesis of LOH.

Testosterone replacement therapy (TRT) is the routine treatment for LOH, and can achieve satisfactory relief of symptoms [5, 10, 11]. However, TRT has contraindications and side effects that cannot be ignored. TRT is not recommended for men with prostate cancer, breast cancer, severe chronic cardiac failure, a hematocrit >0.54%, or those with an active desire to have children [12–15]. In addition, individual differences in the physiological requirements for testosterone often make it difficult for exogenous supplementation to meet the needs of individual patients [16]. Consequently, alternative approaches to increase endogenous testosterone without the disadvantages of TRT need to be developed.

In recent years, senescent cells have emerged as therapeutic target in age-related diseases. This is because senescent cells can disrupt normal tissue function and composition by secreting pro-inflammatory and matrix-degrading molecules, which is known as the senescence-associated secretory phenotype (SASP) [17–19]. Research in animals over the past decade has demonstrated that selective elimination of senescent cells alleviates several age-related diseases and even prolongs lifespan [20–23]. Accordingly, targeting senescent Leydig cells is an attractive therapeutic strategy for LOH.

FOXO4-DRI is a peptide antagonist designed to block the interaction of forkhead box O 4 (FOXO4) and p53 [24]. Using ionizing radiation-induced senescent IMR90 cells, it was observed that FOXO4 maintains senescent cell viability by targeting p53 to the nucleus and preventing it from inducing apoptosis. Disrupting the p53-FOXO4 interaction using FOXO4-DRI caused p53 to be excluded from nucleus and directed to mitochondria for induction of apoptosis, ultimately eliminating the senescent cells. Therefore, the present study was designed to determine the pattern of FOXO4 expression in human testes and its role in Leydig cell senescence. We further sought to use an aged mice model to assess the ability of FOXO4-DRI to eliminate senescent Leydig cells and alleviate testosterone secretion insufficiency.

## RESULTS

### FOXO4 is specifically expressed in Leydig cells within human testes

**Figure 1:**
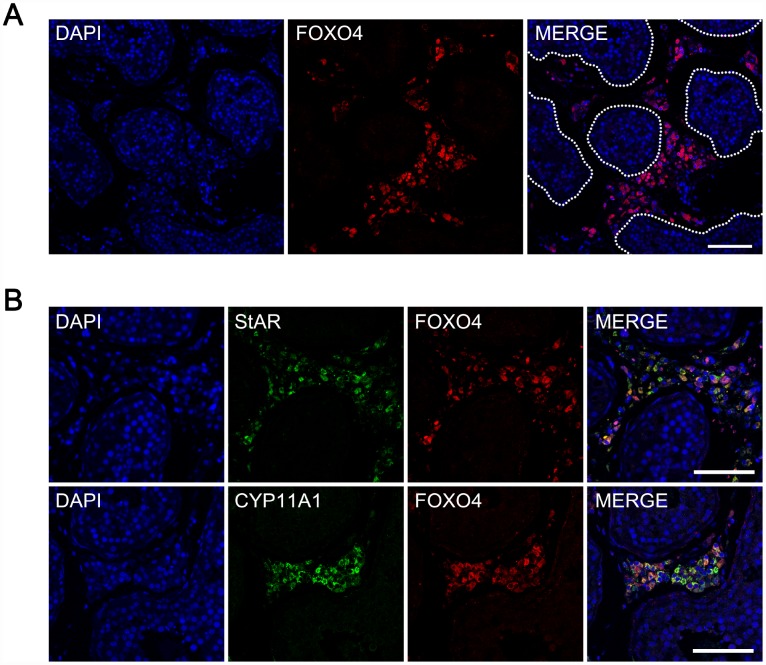
**Expression of FOXO4 in human testes detected by immunofluorescent staining.** (**A**) FOXO4 was expressed in the interstitial or peritubular cells of human testes, but not within the seminiferous tubules. (**B**) FOXO4^+^ cells expressing the Leydig cell markers StAR and CYP11A1. Scale bar: 100 μm.

To evaluate the pattern of FOXO4 expression, tissue sections of human testes were immunofluorescently stained and then photographed using confocal microscopy. FOXO4 expression was detected in the interstitial or peritubular cells, but not within the seminiferous tubules (Figure 1A). Additionally, the FOXO4^+^ cells expressed the Leydig cell markers StAR and CYP11A1, as confirmed by triple immunofluorescent staining (Figure 1B). These results indicate that FOXO4 is specifically expressed in Leydig cells within human testes.

### FOXO4 shows nuclear localization in elderly testicular Leydig cells and is related to decreased testosterone synthesis

**Figure 2:**
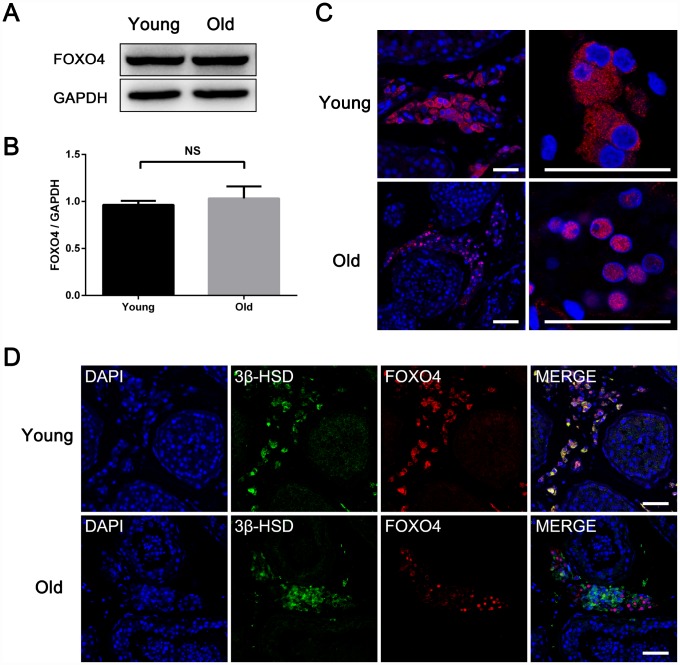
**Differences between testicular FOXO4 expression in young (< 30 years old) and old (≥ 65 years old) men.** (**A**, **B**) Western blotting revealing no significant difference in FOXO4 protein levels between young and old men (n=6). (**C**) Immunofluorescent staining showing that FOXO4 localizes predominantly in the cytoplasm of Leydig cells in young men, but in the nucleus in old men. Scale bar: 40 μm. (**D**) Immunofluorescent staining of FOXO4 and the rate-limiting testosterone synthetic enzyme 3β-HSD showing that nuclear-FOXO4^+^ Leydig cells express less 3β-HSD than nuclear-FOXO4^-^ Leydig cells in testes from the elderly. Scale bar: 50 μm. Data depict the mean ± SD. NS, nonsignificant, *P<0.05.

To explore the role of FOXO4 in the process Leydig cell senescence, we compared expression of FOXO4 in testes from young (< 30 years old) [5] and old (≥ 65 years old, according to the WHO criteria for elderly) males. Western blot analysis revealed no significant difference in FOXO4 levels between the two groups (Figure 2A and 2B). Interestingly, however, FOXO4 was predominantly localized in the cytoplasm of Leydig cells from young men, but in the nucleus in old men (Figure 2C). As FOXO4 is a transcription factor, this nuclear localization indicates higher transcriptional regulatory activity, suggesting FOXO4 activity is involved in Leydig cells senescence. We also found that in elderly testes, nuclear-FOXO4^+^ Leydig cells expressed less 3β-hydroxysteroid dehydrogenase (3β-HSD), the rate-limiting enzyme in testosterone synthesis, than nuclear-FOXO4^-^ Leydig cells (Figure 2D). Taken together, these results suggest that nuclear localization of FOXO4 may contribute to Leydig cell senescence and the downregulation of testosterone synthesis.

### Hydrogen peroxide induces cellular senescence and FOXO4 nuclear translocation in TM3 Leydig cells

**Figure 3:**
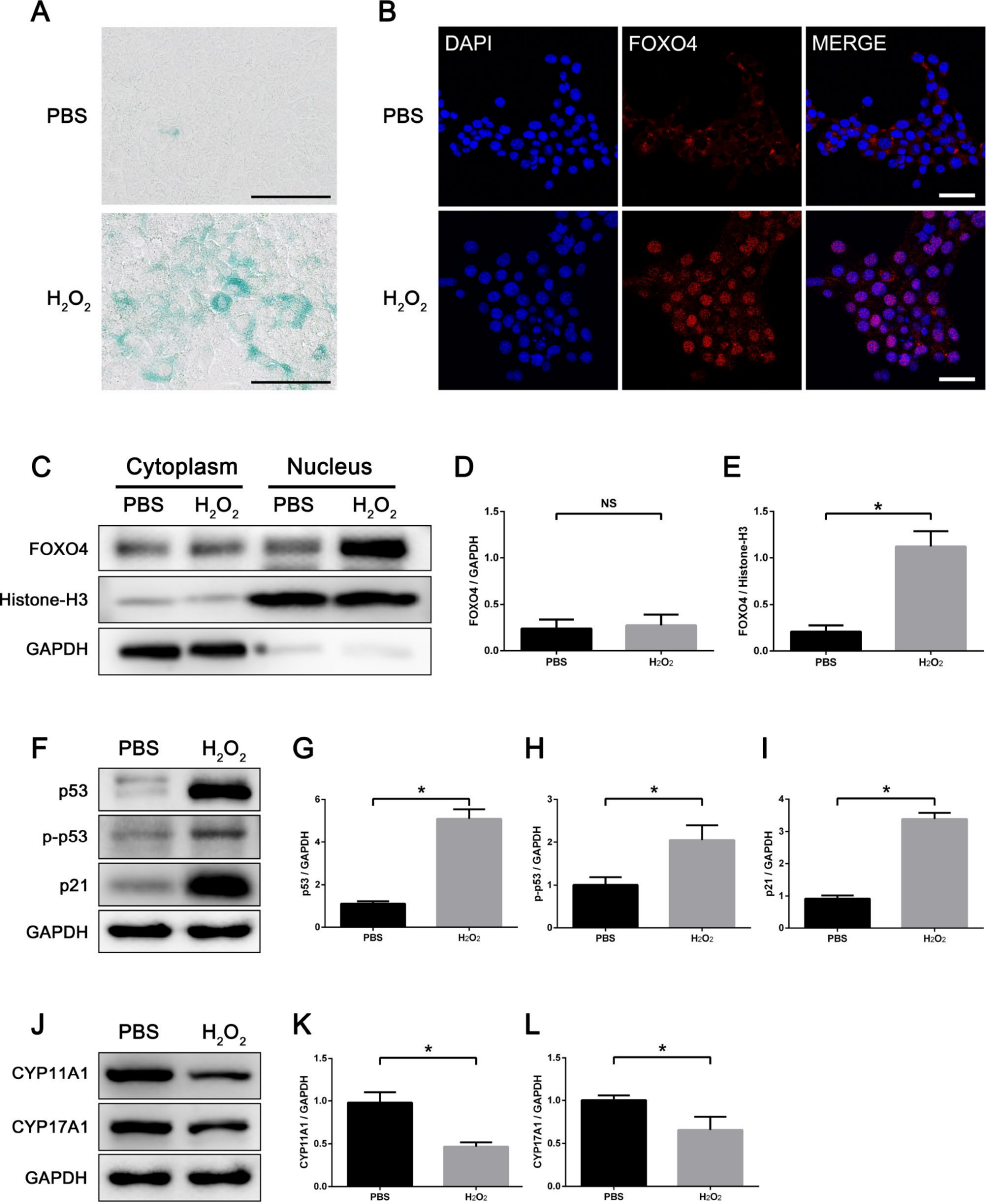
**H_2_O_2_ induces FOXO4 nuclear translocation and cellular senescence in TM3 Leydig cells.** (**A**) SA-β-gal assay showing TM3 Leydig cells with increased SA-β-gal activity after 48 h exposure to 100 μΜ H_2_O_2_ in serum-free medium. Scale bar: 100 μm. (**B**) Immunofluorescent staining showing that H_2_O_2_-induced senescent TM3 Leydig cells express FOXO4 predominantly in the nucleus, while controls express FOXO4 in the cytoplasm. Scale bar: 50 μm. (**C**–**E**) Western blots of separating nuclear and cytoplasmic extracts showing a significant increase in FOXO4 expression in H_2_O_2_-induced senescent TM3 Leydig cells, and FOXO4 concentrated in the nucleus. (**F**–**I**) Western blots of total protein revealing the levels of p53, Ser15-phopho-p53 and p21 are significantly elevated in H_2_O_2_-induced senescent TM3 Leydig cells. (**J**–**L**) Western blots revealing that levels of the testosterone synthesis-related proteins CYP11A1 and CYP17A1 are significantly decreased in H_2_O_2_-induced senescent TM3 Leydig cells. Data presented are representative of three independent experiments. Data depict the mean ± SD. *P<0.05.

To study the role of FOXO4 in regulating Leydig cell senescence *in vitro*, we induced cell senescence in TM3 mouse Leydig cells using hydrogen peroxide (H_2_O_2_). After a 48 h exposure to 100 μM H_2_O_2_ in serum-free medium, TM3 Leydig cells showed an increased senescence-associated β-galactosidase (SA-β-gal) activity (Figure 3A). Consistent with Leydig cells from elderly men, immunofluorescent staining showed FOXO4 predominantly localized in the nucleus of these H_2_O_2_-induced senescent TM3 Leydig cells, but in the cytoplasm of untreated controls (Figure 3B). Western blotting of separating nuclear and cytoplasmic extracts revealed a significant increase in FOXO4 expression in H_2_O_2_-induced senescent TM3 Leydig cells, and confirmed that FOXO4 protein was concentrated in the nucleus (Figure 3C–3E). Western blotting of total protein showed that levels of the cellular senescence-associated pathway proteins p53, Ser15-phospho-p53, and p21, which are signaling molecules downstream of FOXO4, were increased in the senescent cells (Figure 3F–3I), whereas levels of the testosterone synthesis-related proteins CYP11A1 and CYP17A1 were decreased (Figure 3J–3L). These data demonstrate that H_2_O_2_ induces TM3 Leydig cell senescence *in vitro* and that, in these senescent Leydig cells, FOXO4 is translocated to the nucleus and activates a senescence-associated pathway.

### FOXO4 facilitates TM3 Leydig cell senescence and maintains the viability of senescent cells

**Figure 4:**
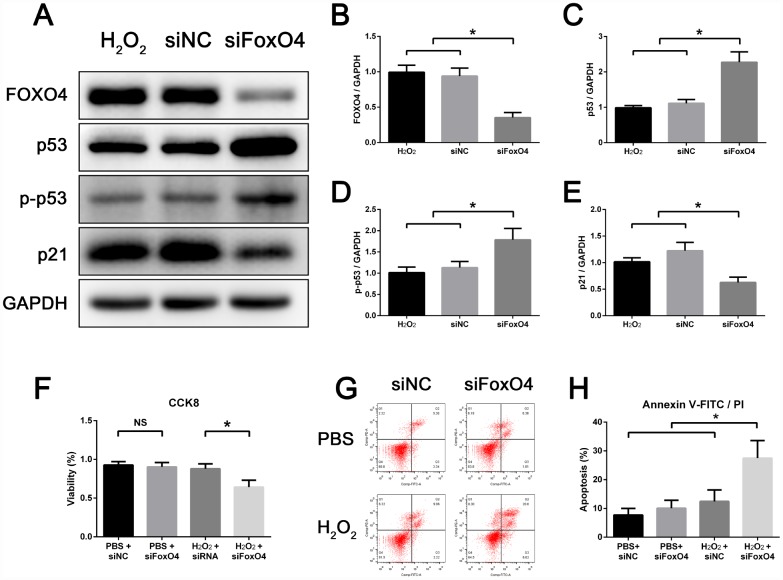
**FOXO4 facilitates TM3 Leydig cell senescence and maintains the viability of senescent cells.** (**A**–**E**) Western blots revealing that, compared to control, FoxO4 knockdown increases protein levels of p53 and Ser15-phospho-p53 but decreases levels of p21 in senescent TM3 Leydig cells. (**F**) CCK8 assays showing that FoxO4 knockdown decreases the viability of senescent TM3 Leydig cells. (**G**, **H**) Annexin V-FITC/PI apoptosis assays showing that FoxO4 knockdown increases the apoptosis rate among senescent TM3 Leydig cells. NC, negative control. Data presented are representative of three independent experiments. Data depict the mean ± SD. *P<0.05. NS, nonsignificant.

To further validate the role of FOXO4 in senescent Leydig cells, we silenced FoxO4 expression using siRNA prior to senescence induction. Following FoxO4 knockdown, the levels of p53 and Ser15-phospho-p53 in senescent TM3 Leydig cells were further increased, but the level of p21 was decreased as compared with their control counterparts (Figure 4A–4E). This indicates that FOXO4 facilitates expression of the p53-target p21 in senescent Leydig cells. FoxO4 knockdown also reduced cell viability (Figure 4F) and increased the incidence of apoptosis among senescent TM3 Leydig cells (Figure 4G and 4H). This suggests that after H_2_O_2_ induction, FOXO4 facilitated Leydig cell senescence while maintaining the viability of senescent cells by repressing their apoptotic response.

### FOXO4-DRI causes nuclear exclusion of active p53 and induces apoptosis in senescent TM3 Leydig cells

**Figure 5:**
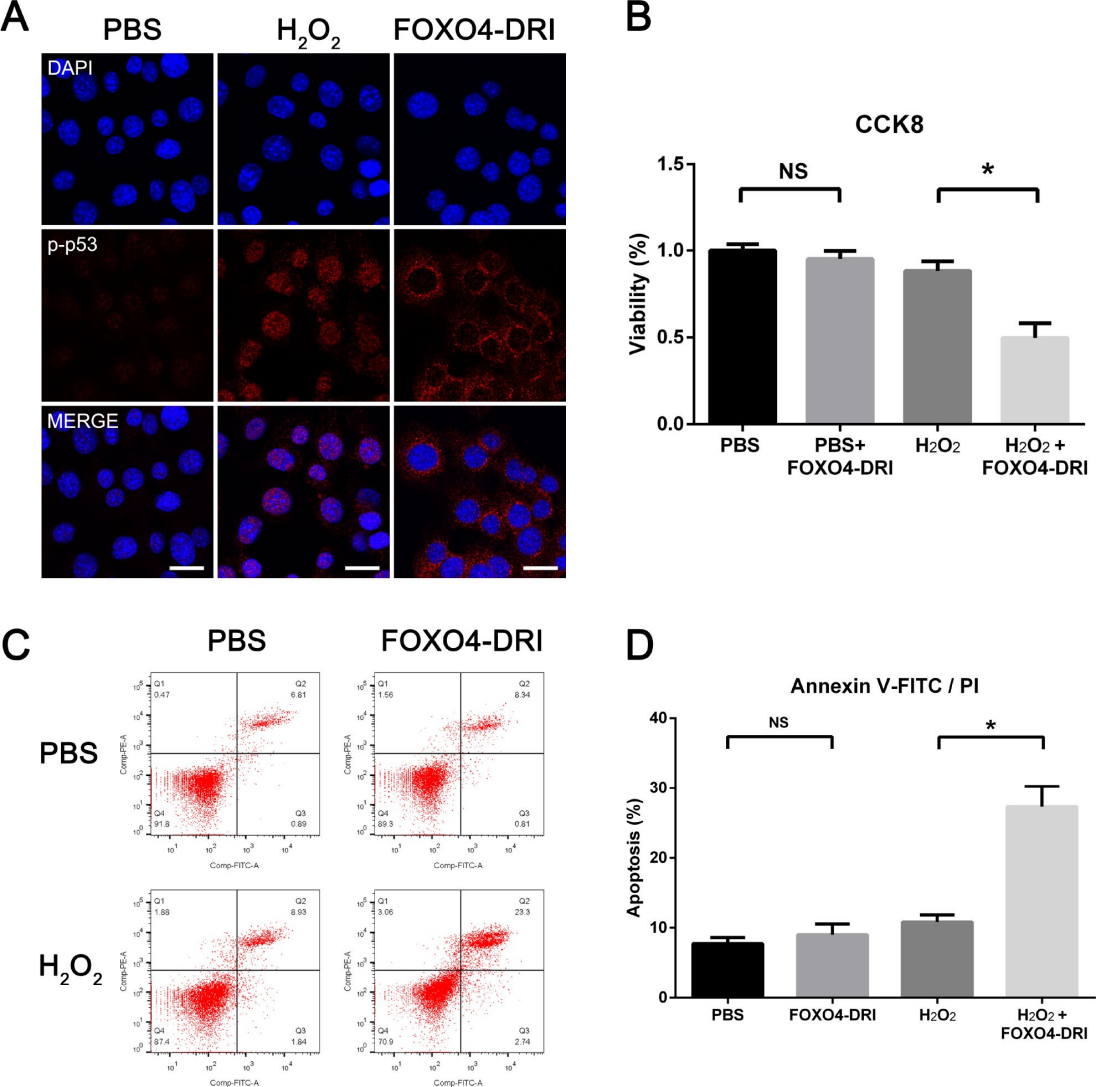
**FOXO4-DRI causes nuclear exclusion of active p53 and induces apoptosis in senescent TM3 Leydig cells.** (**A**) Immunofluorescent staining showing increased levels of Ser15-phospho-p53 in the nucleus of senescent TM3 Leydig cells. Note that Ser15-phospho-p53 foci are excluded from the nucleus after treatment with 25 mM FOXO4-DRI for 3 days. Scale bar: 25 μm. (**B**) CCK8 assays showing that FOXO4-DRI decreases the viability of senescent TM3 Leydig cells. (**C**, **D**) Annexin V-FITC/PI apoptosis assays showing that FOXO4-DRI increases the apoptosis rate among senescent TM3 Leydig cells. Data presented are representative of three independent experiments. Data depict the mean ± SD. *P<0.05. NS, nonsignificant.

Immunofluorescent staining showed elevated levels of Ser15-phospho-p53 in the nucleus of senescent TM3 Leydig cells (Figure 5A). After incubating senescent Leydig cells with 25 mM FOXO4-DRI for 3 days, Ser15-phospho-p53 foci were excluded from the nucleus (Figure 5A). FOXO4-DRI also reduced the viability of senescent as compared to normal TM3 Leydig cells (Figure 5B), and the apoptosis rate increased from 10% to 27% (Figure 5C and 5D). On the other hand, FOXO4-DRI did not show significant toxicity in normal TM3 Leydig cells (Figure 5B–5D), where expression of FOXO4 was low (Figure 3C–3E). These results show that FOXO4-DRI caused nuclear exclusion of Ser15-phospho-p53 and induced apoptosis in senescent TM3 Leydig cells, and that it acted selectively against senescent cells.

### FOXO4-DRI alleviates testosterone secretion insufficiency in naturally aged mice

**Figure 6:**
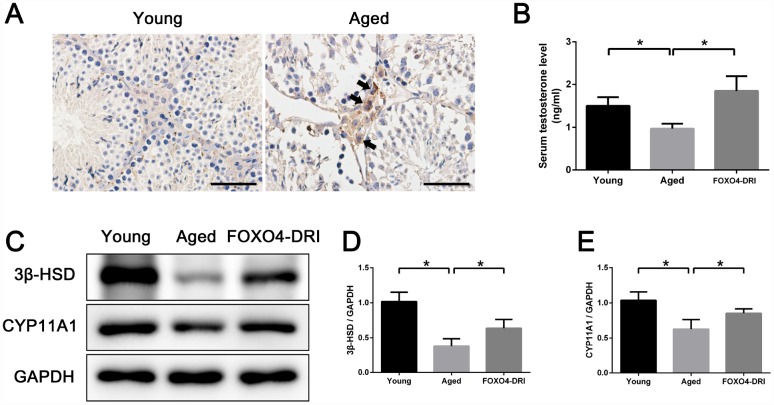
**FOXO4-DRI alleviates testosterone secretion insufficiency in naturally aged mice.** (**A**) Immunohistochemical staining showing that FOXO4 was rarely expressed in young (3 months of age) mice testes, but expressed typically in the Leydig cells of naturally aged (20-24 months of age) mice. In aged mice testes, FOXO4 was predominantly expressed in cytoplasm and showed nuclear localization in some Leydig cells (arrows). Scale bar: 50 μm. (**B**) Serum testosterone levels in naturally aged (20-24 months of age) male mice are lower than in young adult (3 months of age) male mice, but are significantly increased 30 days after FOXO4-DRI treatment (intraperitoneal injection of 5 mg/kg every other day for three administrations). (**C**–**E**) Western blots revealing that FOXO4-DRI treatment increases protein levels of the testosterone synthesis-related proteins 3β-HSD and CYP11A1 in the testes of aged mice. Data depict the mean ± SD; n=6. *P<0.05.

Our finding that FOXO4-DRI targeted senescent TM3 Leydig cells *in vitro* prompted us to investigate its therapeutic effect *in vivo* using naturally aged (20-24 months) male mice as subjects. These aged mice exhibited lower serum testosterone levels than young adult (3 months) mice (Figure 6B), indicating the presence of age-related testosterone secretion insufficiency. Firstly, we detected the expression and location of FOXO4 in mice testes by immunohistochemical staining and found that FOXO4 was rarely expressed in young mice testes, but expressed typically in the Leydig cells of aged mice (Figure 6A). In aged mice testes, FOXO4 was predominantly expressed in cytoplasm and showed nuclear localization in some Leydig cells (Figure 6A). Then the aged mice were intraperitoneally injected with FOXO4-DRI (5 mg/kg) in PBS or PBS alone every other day for three administrations. Thirty days after treatment, there was no significant difference in body weight or testis weight between the FOXO4-DRI-treated mice and their control counterparts (Table 1). Notably, serum testosterone levels were increased in the aged mice treated with FOXO4-DRI (Figure 6B). In line with these changes of serum testosterone, western blot analysis showed that FOXO4-DRI treatment increased levels of both 3β-HSD and CYP11A1 (Figure 6C–6E).

**Table 1:** Body and testis weights of mice.

**Group**	**Nos.**	**Body weight (g)**		**Testis weight (mg)**	**Testis/Body weight ratio (mg/g)**
		**Before**	**After**		
PBS	6	33.57 ± 0.826	34.07 ± 0.881	93.17 ± 1.847	2.742 ± 0.083
FOXO4-DRI	6	32.42 ± 1.129	33.25 ± 0.565	91.25 ± 3.630	2.740 ± 0.086

* Data depict the mean ± SD.

### FOXO4-DRI improves the testicular microenvironment in naturally aged mice

**Figure 7:**
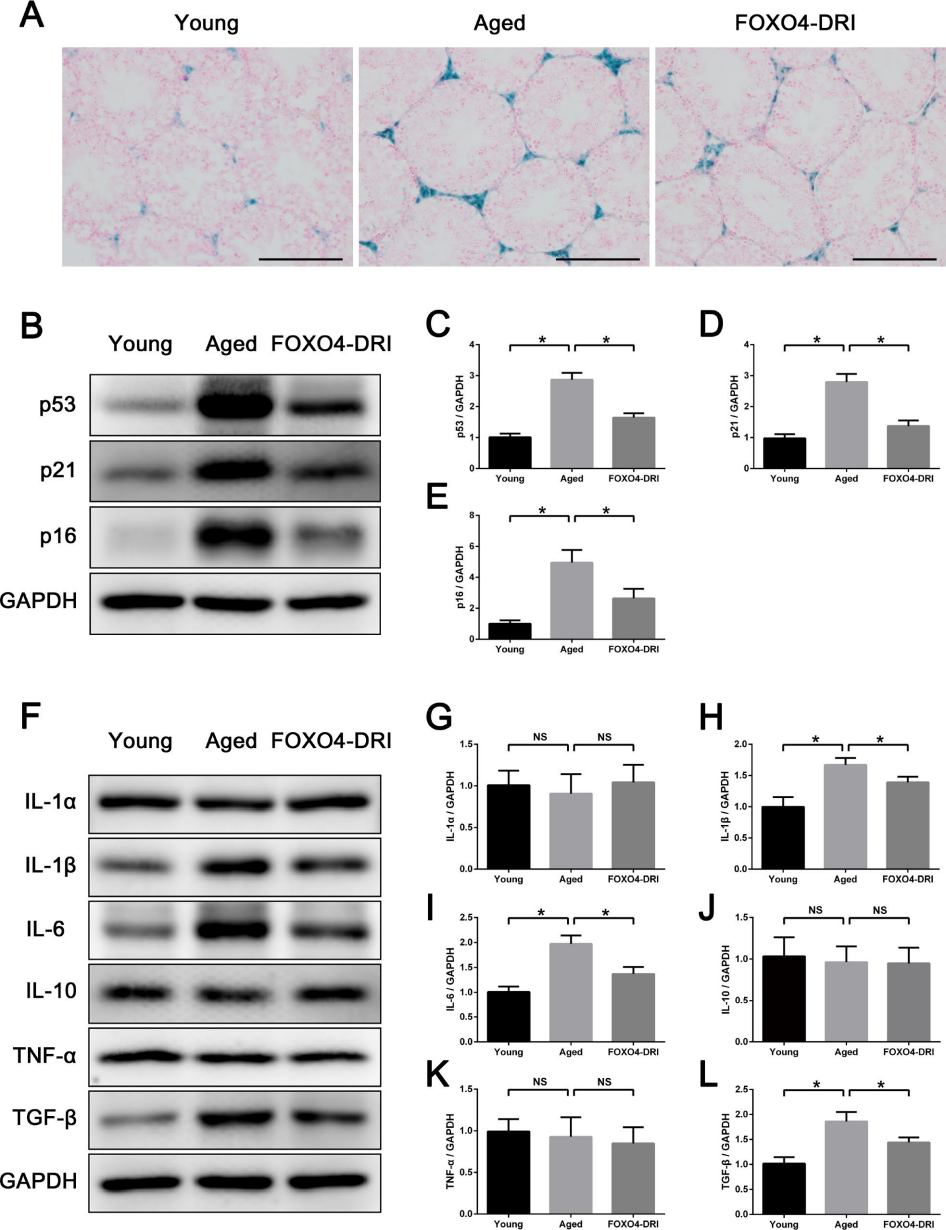
**FOXO4-DRI improves the testicular microenvironment in naturally aged mice.** (**A**) SA-β-gal assay showing that FOXO4-DRI treatment decreases SA-β-gal activity in the testicular interstitium in aged mice. Scale bar: 200 μm. (**B**–**E**) Western blots revealing FOXO4-DRI decreases levels of the senescence-associated proteins p53, p21 and p16 in testes of aged mice. (**F–L**) Western blots revealing that FOXO4-DRI decreases levels of the SASP factors IL-1β, IL-6 and TGF-β in testes of aged mice, but has no effect on testicular levels of IL-1α, IL-10 and TNF-α. Data depict the mean ± SD. n=6. *P<0.05. NS, nonsignificant.

FOXO4-DRI-treated aged testes showed decreased interstitial SA-β-gal activity (Figure 7A) as well as lower levels of senescence-associated proteins p53, p21, and p16 (Figure 7B–7E). This suggests FOXO4-DRI alleviates the testicular senescence phenotype. In addition, to evaluate changes in the testicular microenvironment after FOXO4-DRI treatment, we assessed levels of several common SASP factors in testis tissue, including IL-1α, IL-1β, IL-6, IL-10, TNF-α and TGF-β. Western blotting showed the levels of IL-1β, IL-6 and TGF-β were all decreased in aged mice treated with FOXO4-DRI (Figure 7F–7L). This suggests FOXO4-DRI improves the testicular microenvironment in aged mice, which may be one of the mechanisms by which FOXO4-DRI alleviates testosterone secretion insufficiency.

## DISCUSSION

In the present study, we found that in human testes FOXO4 was specifically expressed in Leydig cells, that FOXO4 showed nuclear translocation in Leydig cells within old testes, and that the nuclear translocation of FOX04 was related to decreased testosterone synthesis. Then using a H_2_O_2_-induced senescent TM3 Leydig cell model, we found that FOXO4 maintains the viability of senescent Leydig cells and that the FOXO4 blocker FOXO4-DRI induces apoptosis in these senescent cells. Finally, we explored the therapeutic effect of FOXO4-DRI in naturally aged mice, and demonstrated that FOXO4-DRI alleviates age-related testosterone secretion insufficiency and improves the testicular microenvironment. These findings suggest the potential usefulness of FOXO4-DRI for the treatment of male LOH.

Senescent cells have recently emerging as a therapeutic target for treating age-related diseases. Existing anti-senescence compounds are mainly anticancer agents, such as ATB-737, navitoclax, and quercetin. ATB-737 and navitoclax are pan-BCL inhibitors targeting the BCL-2/W/XL family of anti-apoptotic guardians [25, 26], while quercetin is a natural product with anticancer properties that acts on various pathways implicated in diverse biological processes [27]. However, these anticancer agents do not selectively kill senescent cells and have only a narrow safe concentration range. As a result, some normal cell populations are likely affected when eliminating senescent cells, and their use is limited by their cytotoxic side effects.

FOXO4-DRI is a cell-permeable peptide comprising part of the p53-interaction domain of FOXO4. It can compete with endogenous FOXO4 for p53, thereby disrupting the FOXO4-p53 interaction [24]. Previous research has shown FOXO4-DRI to be selective for ionizing radiation-induced senescent IMR90 cells and safe to normal cells [24]. In the present study, we obtained similar results in H_2_O_2_-induced senescent TM3 Leydig cells. Our data show that FOXO4-DRI caused nuclear exclusion of active p53 in senescent TM3 Leydig cells. Nuclear exclusion of p53 reportedly leads to translocation of p53 to mitochondria and transcription-independent induction of apoptosis [28], which may explain the therapeutic effect of FOXO4-DRI.

The results of our *in vitro* experiments showed that FOXO4-DRI does not affect the viability of normal Leydig cells. There are two explanations for this result. First, FOXO4 localizes predominantly in the cytoplasm of normal Leydig cells, as shown by both our *in vitro* and *in vivo* findings, while it is within the nucleus that FOXO4-DRI interferes with the FOXO4-p53 interaction [24]. Second, young FoxO4-null mice show no reproductive system impairment [29], indicating that FOXO4 does not play an important role in normal Leydig cells.

Despite a lack of direct evidence showing that FOXO4-DRI can eliminate senescent Leydig cells in aged mice, we observed that FOXO4-DRI treatment alleviates the testicular senescence phenotype, improves the testicular microenvironment, and increases serum testosterone levels, all of which could contribute to the therapeutic effects of FOXO4-DRI. In that context, it is noteworthy that optimal function of stem cells depends on their highly specialized microenvironment, or niche [30, 31], and SASP factors deleteriously affect stem cells by altering this niche [32, 33]. We speculate that by eliminating senescent cells and improving the microenvironment, FOXO4-DRI activates the proliferation or differentiation of stem Leydig cells, serving as Leydig cell progenitors [34], which in turn leads to improved testosterone secretion. This speculation will require testing in future studies.

FOXO4-DRI was systemically administrated in both our study and that of the compound designer [24]. Although it has been demonstrated that FOXO4-DRI has a strong preference for targeting senescent cells [24], because different organs or tissues have different drug sensitivities, the safety and tolerability of FOXO4- DRI must be considered. According to the Human Protein Atlas Database (https://www.proteinatlas.org/), in humans FOXO4 protein is expressed only in testis, placenta and muscle. During treatment, therefore, special attention must be paid to muscle damage, especially cardiotoxicity. Due to technical limitations, we could not address that issue in the present study. In future translational studies, testicular local injection or biomaterial-mediated targeted administration may be a better choice to avoid the disadvantages of systemic administration.

Our study had two main limitations. First, the exact mechanism by which FOXO4-DRI increased serum testosterone levels was not elucidated. Improved function of surviving Leydig cells or differentiation of stem Leydig cells may be involved. Second, no attempt was made to assess when the increase in serum testosterone occurred or for how long the effect lasted. Long-term and systematic evaluation after FOXO4-DRI treatment is needed in further studies.

In summary, our study revealed that FOXO4-DRI alleviates age-related testosterone secretion insufficiency in aged mice. Moreover, it caused nuclear p53 exclusion and induced apoptosis selectively in senescent Leydig cells by disrupting the FOXO4-p53 interaction, which may be a critical mechanism underlying its therapeutic effect. These findings shed new light on a potential treatment for LOH.

## MATERIALS AND METHODS

### Human testes samples

Fourteen testes specimens were obtained from brain-dead donors (six young males, aged 22 to 30) and prostate cancer patients undergoing surgical castration (eight elderly males, aged 66 to 87) at the First Affiliated Hospital of Sun Yat-sen University. Written informed consent was obtained from each subject or their family members. The protocol applied in this study conformed to the ethical guidelines of the Helsinki Declaration and was approved by the Institutional Review Board of the First Affiliated Hospital of Sun Yat-sen University.

### Immunofluorescence and immunohistochemistry

Testis tissues or TM3 cells were fixed and processed for immunofluorescent or immunohistochemical staining as described previously [35]. The primary antibodies included anti-FOXO4 (ab128908, 1:100; Abcam, USA), anti-FOXO4 (sc-373877, 1:100; Santa Cruz, USA), anti-StAR (#8449, 1:100; Cell Signaling Technology, USA), anti-CYP11A1 (GTX56293, 1:100; Gene Tex, USA), anti-3β-HSD (sc-100466, 1:100; Santa Cruz, USA), and anti-Ser15-phospho-p53 (ab1431, 1:100; Abcam, USA) antibodies. Images were captured using a Leica confocal microscope.

### Western blot analysis

Total protein was extracted from tissues or cells using RIPA lysis buffer (CW2333, CWBIO, China) containing proteinase and phosphatase inhibitors. Nuclear and cytoplasmic protein of TM3 cells was extracted using NE-PER Nuclear and Cytoplasmic Extraction Reagents (78833, Thermo Fisher Scientific, USA) according to the manufacturer’s instructions. Western blot analysis was conducted as previously described [35]. The primary antibodies included anti-FOXO4 (ab128908, 1:1000; Abcam, USA), anti-p53 (ab26, 1:200; Abcam, USA), anti- Ser15-phospho-p53 (ab1431, 1:500; Abcam, USA), anti-p21 (ab188224, 1:1000; Abcam, USA), anti-p16 (MAB2416, 1:500; Abnova, USA), anti-3β-HSD (sc-515120, 1:200; Santa Cruz, USA), anti-CYP11A1 (GTX56293, 1:500; Gene Tex, USA), anti-CYP17A1 (ab125022, 1:1000; Abcam, USA), anti-IL-1α (16764-1-AP, 1:500; Proteintech, USA), anti-IL-1β (ab9722, 0.2 μg/ml; Abcam, USA), anti-IL-6 (ab9324, 0.4 μg/ml; Abcam, USA), anti-IL-10 (DF6894, 1:500; Affinity, USA), anti-TNF-α (17590-1-AP, 1:500; Proteintech, USA), anti-TGF-β (18978-1-AP, 1:500; Proteintech, USA) and anti-GAPDH (T0004, 1:5000; Affinity, USA) antibodies. GAPDH was used as the control.

### Cell culture

The TM3 mouse Leydig cell line was purchased from the Cell Bank of Chinese Academy of Sciences (Shanghai, China). The cells were cultured in DMEM/F-12 medium (11320033, Gibco, USA) supplemented with 5% horse serum and 2.5% fetal bovine serum at 37°C under an atmosphere of 5% CO_2_.

### SA-β-gal assay

SA-β-gal activity was measured in cells or frozen tissues using a Senescence β-Galactosidase Staining Kit (C0602, Beyotime, China) according to the manufacturer’s instructions. Frozen tissues were counterstained with Nuclear Fast Red after the SA-β-gal staining. Images were captured with an Olympus inverted microscope.

### Cell transfection

FoxO4 siRNA and negative control siRNA were obtained from Viewsolid (Beijing, China). These siRNA oligos were transfected into cells at 50% confluence for 6 h using Lipofectamine 3000 Kit (L3000015, Thermo Fisher Scientific, USA), following the manufacturer’s instructions. The proteins were extracted 48 h after transfection. The sense and antisense sequences of FoxO4 siRNA were 5′-GGCUCCUACACUUCUGUUATT-3′ and 5′-UAACAGAAGUGUAGGAGCCTT-3′, respectively.

### Cell viability assay

Cell viability was assessed using Cell Counting Kit-8 (CCK8; CK04, Dojindo, Japan) according to the manufacturer’s instructions. Briefly, TM3 cells were seeded into 96-well plates at a density of 5×10^3^ cells/well. After different treatments, 10 μl of CCK8 solution were added to each well and incubated for 2 h at 37°C. Absorbance at 450 nm was then measured using a microplate reader.

### Cell apoptosis assays

Cell apoptosis was assessed using an Annexin V-FITC/PI Apoptosis Detection Kit (CW2574, CWBIO, China) according to the manufacturer’s instructions. Briefly, TM3 cells were harvested after different treatments and mixed with 5 μl of Annexin V-FITC and 10 μl of 20 μg/ml PI reagent. The cells were then incubated for 15 min at room temperature with no light. After then adding 400 μl of PBS, the samples were subjected to flow cytometric analysis to detect cell apoptosis levels. The apoptotic index was calculated as the sum of the FITC-Annexin V-positive/PI-negative (early apoptosis) and FITC-Annexin V-positive/PI-positive (late apoptosis) cell populations.

### Animals

Twenty naturally aged male C57BL/6 mice (20-24 months old) and another 10 young adult male C57BL/6 mice (3 months old) from the Laboratory Animal Center of Sun Yat-sen University were used in these experiments. All experimental procedures involving animals were approved by the Institutional Animal Care and Use Committee of Sun Yat-sen University.

### Testosterone concentration assay

Mouse blood samples were drawn 60 minutes at room temperature, and serum was obtained after centrifugation (3000 rpm, 15 min, 4°C). The serum testosterone concentrations were measured by electrochemiluminescence immunoassay using Elecsys Testosterone II (05200067190, Roche, Germany) according to the manufacturer’s instructions.

### Statistical analysis

Statistical analyses were performed using IBM SPSS Statistics 23.0 (IBM, USA). All data are expressed as the mean ± standard deviation (SD). Student’s *t* tests for comparisons between two groups and one-way analysis of variance followed by a Student-Newman-Keuls post hoc test for multiple comparisons were used as appropriate. Values of P < 0.05 were considered statistically significant.

## References

[1] Wu FC, Tajar A, Beynon JM, Pye SR, Silman AJ, Finn JD, O’Neill TW, Bartfai G, Casanueva FF, Forti G, Giwercman A, Han TS, Kula K, et al, and EMAS Group. Identification of late-onset hypogonadism in middle-aged and elderly men. N Engl J Med. 2010; 363:123–35. 10.1056/NEJMoa0911101 20554979

[2] Tajar A, Forti G, O’Neill TW, Lee DM, Silman AJ, Finn JD, Bartfai G, Boonen S, Casanueva FF, Giwercman A, Han TS, Kula K, Labrie F, et al, and EMAS Group. Characteristics of secondary, primary, and compensated hypogonadism in aging men: evidence from the European Male Ageing Study. J Clin Endocrinol Metab. 2010; 95:1810–18. 10.1210/jc.2009-1796 20173018

[3] Xu W, Zhu Q, Zhang B, Liu S, Dai X, Gao C, Gao L, Cui Y. Protective effect of calretinin on testicular Leydig cells via the inhibition of apoptosis. Aging (Albany NY). 2017; 9:1269–79. 10.18632/aging.101226 28437248 PMC5425126

[4] Erenpreiss J, Fodina V, Pozarska R, Zubkova K, Dudorova A, Pozarskis A. Prevalence of testosterone deficiency among aging men with and without morbidities. Aging Male. 2019; [Epub ahead of print]. 10.1080/13685538.2019.1621832 31156000

[5] Huhtaniemi I, Forti G. Male late-onset hypogonadism: pathogenesis, diagnosis and treatment. Nat Rev Urol. 2011; 8:335–44. 10.1038/nrurol.2011.47 21502974

[6] Pye SR, Huhtaniemi IT, Finn JD, Lee DM, O’Neill TW, Tajar A, Bartfai G, Boonen S, Casanueva FF, Forti G, Giwercman A, Han TS, Kula K, et al, and EMAS Study Group. Late-onset hypogonadism and mortality in aging men. J Clin Endocrinol Metab. 2014; 99:1357–66. 10.1210/jc.2013-2052 24423283

[7] Ye L, Li X, Li L, Chen H, Ge RS. Insights into the Development of the Adult Leydig Cell Lineage from Stem Leydig Cells. Front Physiol. 2017; 8:430. 10.3389/fphys.2017.00430 28701961 PMC5487449

[8] Chen H, Liu J, Luo L, Zirkin BR. Dibutyryl cyclic adenosine monophosphate restores the ability of aged Leydig cells to produce testosterone at the high levels characteristic of young cells. Endocrinology. 2004; 145:4441–46. 10.1210/en.2004-0639 15231695

[9] Midzak AS, Chen H, Papadopoulos V, Zirkin BR. Leydig cell aging and the mechanisms of reduced testosterone synthesis. Mol Cell Endocrinol. 2009; 299:23–31. 10.1016/j.mce.2008.07.016 18761053

[10] Srinivas-Shankar U, Roberts SA, Connolly MJ, O’Connell MD, Adams JE, Oldham JA, Wu FC. Effects of testosterone on muscle strength, physical function, body composition, and quality of life in intermediate-frail and frail elderly men: a randomized, double-blind, placebo-controlled study. J Clin Endocrinol Metab. 2010; 95:639–50. 10.1210/jc.2009-1251 20061435

[11] Corona G, Rastrelli G, Morgentaler A, Sforza A, Mannucci E, Maggi M. Meta-analysis of Results of Testosterone Therapy on Sexual Function Based on International Index of Erectile Function Scores. Eur Urol. 2017; 72:1000–11. 10.1016/j.eururo.2017.03.032 28434676

[12] Basaria S. Male hypogonadism. Lancet. 2014; 383:1250–63. 10.1016/S0140-6736(13)61126-5 24119423

[13] Bhattacharya RK, Bhattacharya SB. Late-Onset Hypogonadism and Testosterone Replacement in Older Men. Clin Geriatr Med. 2015; 31:631–44. 10.1016/j.cger.2015.07.001 26476121

[14] Moss JL, Crosnoe LE, Kim ED. Effect of rejuvenation hormones on spermatogenesis. Fertil Steril. 2013; 99:1814–20. 10.1016/j.fertnstert.2013.04.003 23663992

[15] Corona G, Rastrelli G, Maggi M. Diagnosis and treatment of late-onset hypogonadism: systematic review and meta-analysis of TRT outcomes. Best Pract Res Clin Endocrinol Metab. 2013; 27:557–79. 10.1016/j.beem.2013.05.002 24054931

[16] Kempenaers B, Peters A, Foerster K. Sources of individual variation in plasma testosterone levels. Philos Trans R Soc Lond B Biol Sci. 2008; 363:1711–23. 10.1098/rstb.2007.0001 18048297 PMC2367619

[17] Childs BG, Durik M, Baker DJ, van Deursen JM. Cellular senescence in aging and age-related disease: from mechanisms to therapy. Nat Med. 2015; 21:1424–35. 10.1038/nm.4000 26646499 PMC4748967

[18] Childs BG, Gluscevic M, Baker DJ, Laberge RM, Marquess D, Dananberg J, van Deursen JM. Senescent cells: an emerging target for diseases of ageing. Nat Rev Drug Discov. 2017; 16:718–35. 10.1038/nrd.2017.116 28729727 PMC5942225

[19] van Deursen JM. The role of senescent cells in ageing. Nature. 2014; 509:439–46. 10.1038/nature13193 24848057 PMC4214092

[20] van Deursen JM. Senolytic therapies for healthy longevity. Science. 2019; 364:636–37. 10.1126/science.aaw1299 31097655 PMC6816502

[21] Xu M, Pirtskhalava T, Farr JN, Weigand BM, Palmer AK, Weivoda MM, Inman CL, Ogrodnik MB, Hachfeld CM, Fraser DG, Onken JL, Johnson KO, Verzosa GC, et al. Senolytics improve physical function and increase lifespan in old age. Nat Med. 2018; 24:1246–56. 10.1038/s41591-018-0092-9 29988130 PMC6082705

[22] Baker DJ, Childs BG, Durik M, Wijers ME, Sieben CJ, Zhong J, Saltness RA, Jeganathan KB, Verzosa GC, Pezeshki A, Khazaie K, Miller JD, van Deursen JM. Naturally occurring p16(Ink4a)-positive cells shorten healthy lifespan. Nature. 2016; 530:184–89. 10.1038/nature16932 26840489 PMC4845101

[23] Jeon OH, Kim C, Laberge RM, Demaria M, Rathod S, Vasserot AP, Chung JW, Kim DH, Poon Y, David N, Baker DJ, van Deursen JM, Campisi J, Elisseeff JH. Local clearance of senescent cells attenuates the development of post-traumatic osteoarthritis and creates a pro-regenerative environment. Nat Med. 2017; 23:775–81. 10.1038/nm.4324 28436958 PMC5785239

[24] Baar MP, Brandt RMC, Putavet DA, Klein JDD, Derks KWJ, Bourgeois BRM, Stryeck S, Rijksen Y, van Willigenburg H, Feijtel DA, van der Pluijm I, Essers J, van Cappellen WA, et al. Targeted Apoptosis of Senescent Cells Restores Tissue Homeostasis in Response to Chemotoxicity and Aging. Cell. 2017; 169:132–47. 10.1016/j.cell.2017.02.031 28340339 PMC5556182

[25] Yosef R, Pilpel N, Tokarsky-Amiel R, Biran A, Ovadya Y, Cohen S, Vadai E, Dassa L, Shahar E, Condiotti R, Ben-Porath I, Krizhanovsky V. Directed elimination of senescent cells by inhibition of BCL-W and BCL-XL. Nat Commun. 2016; 7:11190. 10.1038/ncomms11190 27048913 PMC4823827

[26] Chang J, Wang Y, Shao L, Laberge RM, Demaria M, Campisi J, Janakiraman K, Sharpless NE, Ding S, Feng W, Luo Y, Wang X, Aykin-Burns N, et al. Clearance of senescent cells by ABT263 rejuvenates aged hematopoietic stem cells in mice. Nat Med. 2016; 22:78–83. 10.1038/nm.4010 26657143 PMC4762215

[27] Zhu Y, Tchkonia T, Pirtskhalava T, Gower AC, Ding H, Giorgadze N, Palmer AK, Ikeno Y, Hubbard GB, Lenburg M, O’Hara SP, LaRusso NF, Miller JD, et al. The Achilles’ heel of senescent cells: from transcriptome to senolytic drugs. Aging Cell. 2015; 14:644–58. 10.1111/acel.12344 25754370 PMC4531078

[28] Leu JI, Dumont P, Hafey M, Murphy ME, George DL. Mitochondrial p53 activates Bak and causes disruption of a Bak-Mcl1 complex. Nat Cell Biol. 2004; 6:443–50. 10.1038/ncb1123 15077116

[29] Hosaka T, Biggs WH 3rd, Tieu D, Boyer AD, Varki NM, Cavenee WK, Arden KC. Disruption of forkhead transcription factor (FOXO) family members in mice reveals their functional diversification. Proc Natl Acad Sci USA. 2004; 101:2975–80. 10.1073/pnas.0400093101 14978268 PMC365730

[30] Segel M, Neumann B, Hill MF, Weber IP, Viscomi C, Zhao C, Young A, Agley CC, Thompson AJ, Gonzalez GA, Sharma A, Holmqvist S, Rowitch DH, et al. Niche stiffness underlies the ageing of central nervous system progenitor cells. Nature. 2019; 573:130–34. 10.1038/s41586-019-1484-9 31413369 PMC7025879

[31] Conboy IM, Conboy MJ, Wagers AJ, Girma ER, Weissman IL, Rando TA. Rejuvenation of aged progenitor cells by exposure to a young systemic environment. Nature. 2005; 433:760–64. 10.1038/nature03260 15716955

[32] Goodell MA, Rando TA. Stem cells and healthy aging. Science. 2015; 350:1199–204. 10.1126/science.aab3388 26785478

[33] Brack AS, Conboy MJ, Roy S, Lee M, Kuo CJ, Keller C, Rando TA. Increased Wnt signaling during aging alters muscle stem cell fate and increases fibrosis. Science. 2007; 317:807–10. 10.1126/science.1144090 17690295

[34] Chen H, Wang Y, Ge R, Zirkin BR. Leydig cell stem cells: Identification, proliferation and differentiation. Mol Cell Endocrinol. 2017; 445:65–73. 10.1016/j.mce.2016.10.010 27743991 PMC5346484

[35] Yang Q, Chen X, Zheng T, Han D, Zhang H, Shi Y, Bian J, Sun X, Xia K, Liang X, Liu G, Zhang Y, Deng C. Transplantation of Human Urine-Derived Stem Cells Transfected with Pigment Epithelium-Derived Factor to Protect Erectile Function in a Rat Model of Cavernous Nerve Injury. Cell Transplant. 2016; 25:1987–2001. 10.3727/096368916X691448 27075964

